# Primary Ewing Sarcoma of the Lung: Integrative Analysis of Clinical, Histopathological, Immunohistochemical, and Cytogenetic Features with Literature Review

**DOI:** 10.1007/s13193-025-02480-9

**Published:** 2026-01-05

**Authors:** Ipsita Dhal, Paramita Rudra Pal, Kirti Rajput, Akhil Kapoor, Anuj Gupta, Ankita Pal, Arvind Suresh, Mayank Tripathi, Ajay Choubey

**Affiliations:** 1 Department of Oncopathology, Mahamana Pandit Madan Mohan Malviya Cancer Centre, Homi Bhabha National Institute, Homi Bhabha cancer hospital, Varanasi, India; 2 Department of Medical Oncology, Mahamana Pandit Madan Mohan Malviya Cancer Centre, Homi Bhabha National Institute, Varanasi, India; 3 Department of Clinical Research Secretariat, Mahamana Pandit Madan Mohan Malviya Cancer Centre, Homi Bhabha National Institute, Varanasi, India; 4 Department of Nuclear Medicine, Mahamana Pandit Madan Mohan Malviya Cancer Centre, Homi Bhabha National Institute, Varanasi, India; 5 Department of Surgical Oncology, Mahamana Pandit Madan Mohan Malviya Cancer Centre, Homi Bhabha National Institute, Varanasi , India; 6 Department of Radiation Oncology, Mahamana Pandit Madan Mohan Malviya Cancer Centre, Homi Bhabha National Institute, Varanasi, India

**Keywords:** Lung, Ewing sarcoma, Immunohistochemistry, Chemotherapy

## Abstract

**Introduction:**

Ewing sarcoma (ES) is a highly aggressive malignant neoplasm characterized by small, round, undifferentiated cells presumed neural crest origin, accounting for approximately 10–15% of all primary bone tumors. While it predominantly involves the skeletal system, ES can also arise in extraosseous soft tissues, including the retroperitoneum, thoracic wall, paravertebral region, and extremities. Primary pulmonary involvement by Ewing sarcoma is exceedingly uncommon, with fewer than 60 cases documented in the literature over the past decade.

**Aim:**

The objective of this study was to analyze the clinicopathological characteristics of primary pulmonary Ewing sarcoma cases diagnosed at our institution, supplemented by a comprehensive review of the existing literature.

**Materials and methods:**

A retrospective study was undertaken to evaluate cases of primary pulmonary Ewing sarcoma (ES) diagnosed at the Department of OncoPathology, MPMMCC, Varanasi, from January 2019 to July 2024. Thirteen histopathologically confirmed cases were identified. Comprehensive demographic and clinical data were retrieved from institutional medical records. Detailed analyses of histomorphological features, and immunohistochemical profiles, and molecular alterations was done. Longitudinal follow-up data were analyzed to assess patient survival and clinical outcomes.

**Results:**

The median age of the study cohort was 24 years, ranging from 8 to 63 years. A male predominance was observed, with 69.23% of cases (n=9) occurring in males and 30.76% (n=4) in females. The majority of tumors (92.3%) measured greater than 10 cm in maximum dimension, a finding that was statistically significant (p = 0.001). Immunohistochemically, NKX2.2, FLI-1, and CD99 demonstrated consistent and robust expression across most cases, serving as reliable diagnostic markers. Four out of six patients show EWSR-1 fusion in cytogenetics study. All patients underwent systemic chemotherapy, with one case subsequently managed with surgical resection. Additionally, three patients received adjuvant radiotherapy. Disease progression was documented in three out of thirteen patients. The mean progression-free survival (PFS) was estimated at 19 months (95% confidence interval [CI]: 12.9–26.1 months), while the mean overall survival (OS) was calculated at 13 months (95% CI: 9.7–23.0 months).

**Conclusion:**

Primary pulmonary Ewing sarcoma should be considered in the differential diagnosis of large pulmonary masses, particularly in the absence of a detectable extrathoracic primary lesion and after the exclusion of more common differential diagnoses. Definitive diagnosis relies on histopathological examination supported by immunohistochemistry, with cytogenetic and molecular analyses such as detection of EWSR1 gene rearrangement playing a critical role in confirming the diagnosis. Optimal patient management necessitates a multidisciplinary team approach to ensure early detection and improved therapeutic outcomes.

**Supplementary Information:**

The online version contains supplementary material available at 10.1007/s13193-025-02480-9.

## Introduction

Ewing sarcoma (EWS) is a rare malignant neuroectodermal tumor that represents 6–8% of primary bone cancers and is the second most common bone tumor in children, adolescents, and young adults [1]. It is rare in patients younger than 5 years and older than 30 years. They are characterized by small, round blue cells, which makes them pathologically similar to other small round cell tumors, and they present with a variety of clinical manifestations. It primarily affects the long bones, pelvis, and ribs. The disease is driven by fusion genes between the FET and ETS families, most commonly the **t(11;22)(q24;q12)** translocation, which creates the **EWSR1-FLI1 fusion** in approximately 85–90% of cases [2].

Extraskeletal Ewing Sarcoma (EEWS) is ten times less common than the skeletal counterpart, with an incidence of 0.4 per million [3]. Its prevalence is bimodal, peaking in people under five and over thirty-five. EEWS patients are typically older than patients with skeletal EWS, and unlike skeletal one, EEWS is not correlated with a patient’s race or sex [4]. Commonest extraosseous sites include the chest wall, paravertebral and gluteal muscles, and retroperitoneal space [5]. Primary pulmonary Ewing sarcoma (PES) is particularly uncommon, with fewer than 60 cases documented since Hammar et al. in 1989 [6]. Due to this rarity, clinical, pathological, and molecular characteristics of PES remain poorly understood. We herein present thirteen cases of primary pulmonary EWS, describing their clinicopathological and immunohistochemical features.

## Materials and methods

A retrospective study was conducted in the Department of Oncopathology, MPMMCC after Institutional Ethical Committee approval (IEC No. 11000698). The waiver of consent was taken for this study as the research did not require direct contact with participants, and utilized anonymized biological samples or data. Thirteen cases of primary EWS of the lung were identified in a period of 4.5 years (January 2019-June 2024). Primary pulmonary Ewing sarcoma was confirmed by excluding extrapulmonary primary tumors through comprehensive imaging, including CT scans, whole-body MRI, and FDG PET-CT. Hematoxylin–eosin-stained sections were reviewed in all cases. Representative paraffin blocks and immunohistochemical slides were available for all cases. Deparaffinized tissue sections were incubated with antibodies directed against NKX2.2, CD99, LCA, Desmin, PanCK, TTF1, Synaptophysin, Chromogranin using the polymeric biotin-free horseradish peroxidase method. Appropriate positive controls were run for all antibodies tested. Clinical and follow-up information was obtained from the Electronic Medical Records of the hospital.

All analyses were conducted using SPSS Software version 30. The data were organized and analyzed using statistical methods to determine the distribution, including proportions, means, medians, and modes. Progression-free survival (PFS) and overall survival (OS) were estimated using the Kaplan–Meier method.

## Results

Between January 2019 and July 2024, a total of 3,353 pulmonary specimens comprising both biopsy and surgically resected tissues were submitted for comprehensive histopathological assessment. Adenocarcinoma constituted the predominant histological subtype, identified in 1,509 cases (44.98%), followed by small cell carcinoma in 801 cases (23.88%) and squamous cell carcinoma in 505 cases (15.06%). The remaining 538 cases (16.05%) encompassed a heterogeneous group of less common neoplasms. Within this subset, 13 cases were classified as primary pulmonary Ewing’s sarcoma, representing 0.39% of the total specimens analyzed.

The cohort of pulmonary Ewing’s sarcoma cases predominantly comprised adults, with 69.2% of patients aged ≥ 18 years, and showed a similar gender distribution, with 69.2% being male. Tumor locations were confined to the pulmonary region, with the majority (10/13; 76.92%) arising within the lung parenchyma and the remainder originating from the pleura. The right lung was involved in 6 cases, while the left lung was affected in 4 cases. Most tumors were large at presentation, with 84.6% measuring more than 10 cm in maximum dimension [Table 1].

**Table 1:** Clinico-demographic and clinical presentation details of patients with primary pulmonary ewing’s sarcoma [*n* = 13]

Case No.	Sex	Age (years)	Site of Involvement	Tumor Dimensions at Diagnosis (cm)	Clinical Presentation
1	M	15	Left lung	14.2 × 9.7	Dyspnea on exertion, dry cough, chest pain
2	M	18	Left lung (upper lobe)	12.1 × 10.1 × 14.2	Left-sided chest pain, intermittent fever, episodic cough
3	F	18	Left lung	18.8 × 15.1 × 24.9	Dyspnea, intermittent fever, hoarseness of voice, generalized weakness
4	M	24	Right lung (middle and lower lobes)	18.3 × 13.5 × 13.2	Right-sided chest and back pain, dyspnea
5	F	33	Right lung (upper and lower lobes)	15.8 × 15.7 × 15.0	Dyspnea
6	M	28	Left basal pleura	16.1 × 9.9	Left-sided chest pain
7	M	63	Right pleura	10.6 × 10.0 × 9.5	Hemoptysis, abdominal pain, dyspnea on exertion
8	M	8	Right lung (lower and middle lobes)	25.0 × 13.0 × 13.0	Intermittent fever, anorexia
9	M	11	Right lung	13.1 × 10.3 × 16.3	Chest pain, intermittent fever and cough
10	M	25	Left lung	19.0 × 18.0 × 16.0	Respiratory distress
11	F	52	Right lung (upper lobe)	8.2 × 5.4 × 5.2	Chest pain, dry cough, dyspnea on exertion, nausea, vomiting, fever, anorexia
12	F	15	Right lung	16.8 × 14.1	Cough, dyspnea, chest pain
13	M	25	Right pleura	Multiple pleural-based lesions in the right lower pleural cavity (largest 6.6 × 4.0)	Dyspnea on exertion, chest pain, fever, dry cough, anorexia

**Fig. 1:**
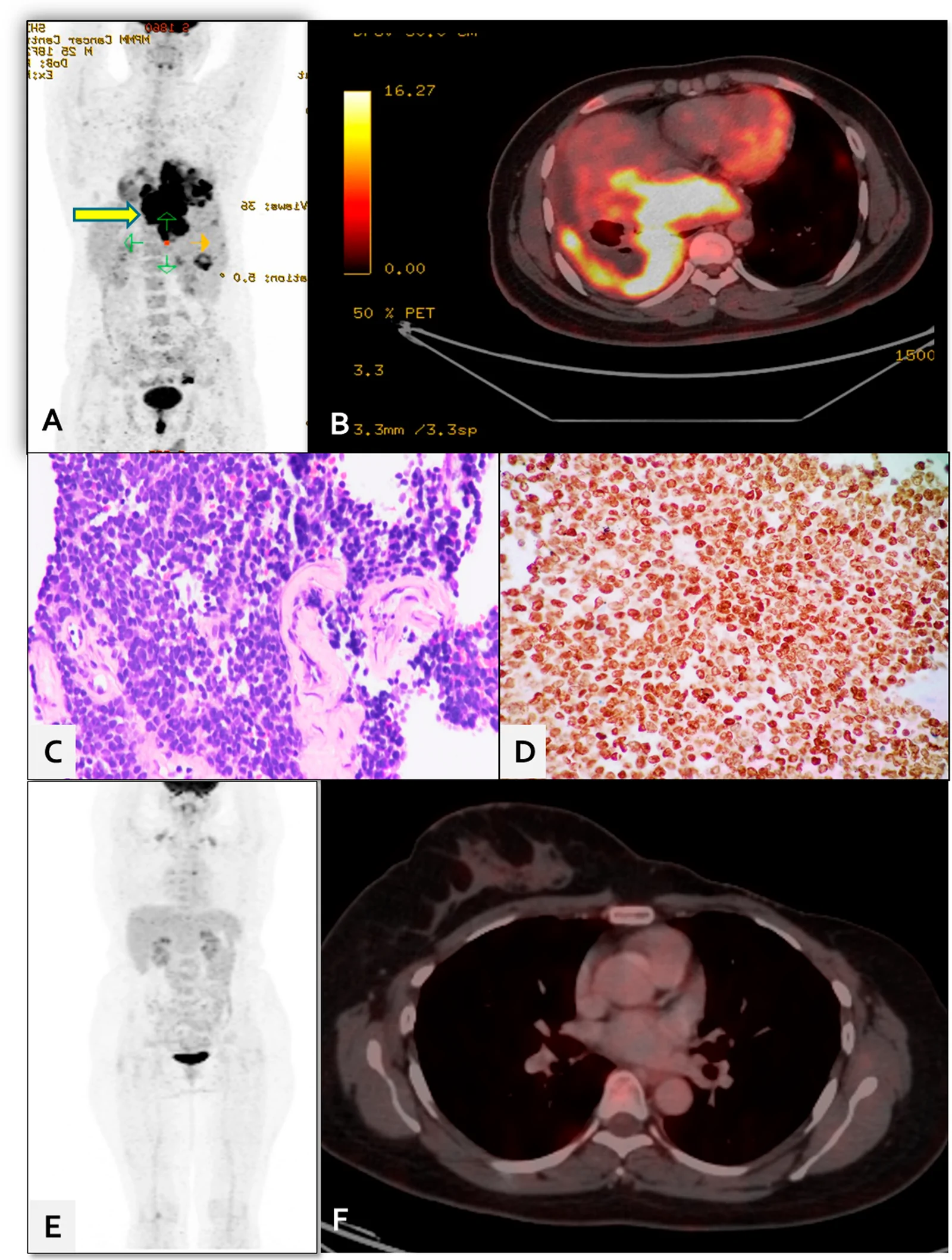
(**A**, **B**) Pre-treatment PET CT scan shows FDG avid multiple irregular pleural based lesion in right lower pleural cavity, largest measuring: 6.6 × 4 × 4 cm, SUVmax 10.53 (arrow). No evidence of metabolically active disease noted elsewhere in the body. (**C**) Biopsy from the mass shows sheets of malignant small round cells, [H&E, 40X]. (**D**) On immunohistochemistry, tumor cells are diffusely positive for NKX2.2. (**E**, **F**) Post treatment [EFT 2001 Induction (VAC and VIME)] response PET CT scan shows complete resolution

**Fig. 2:**
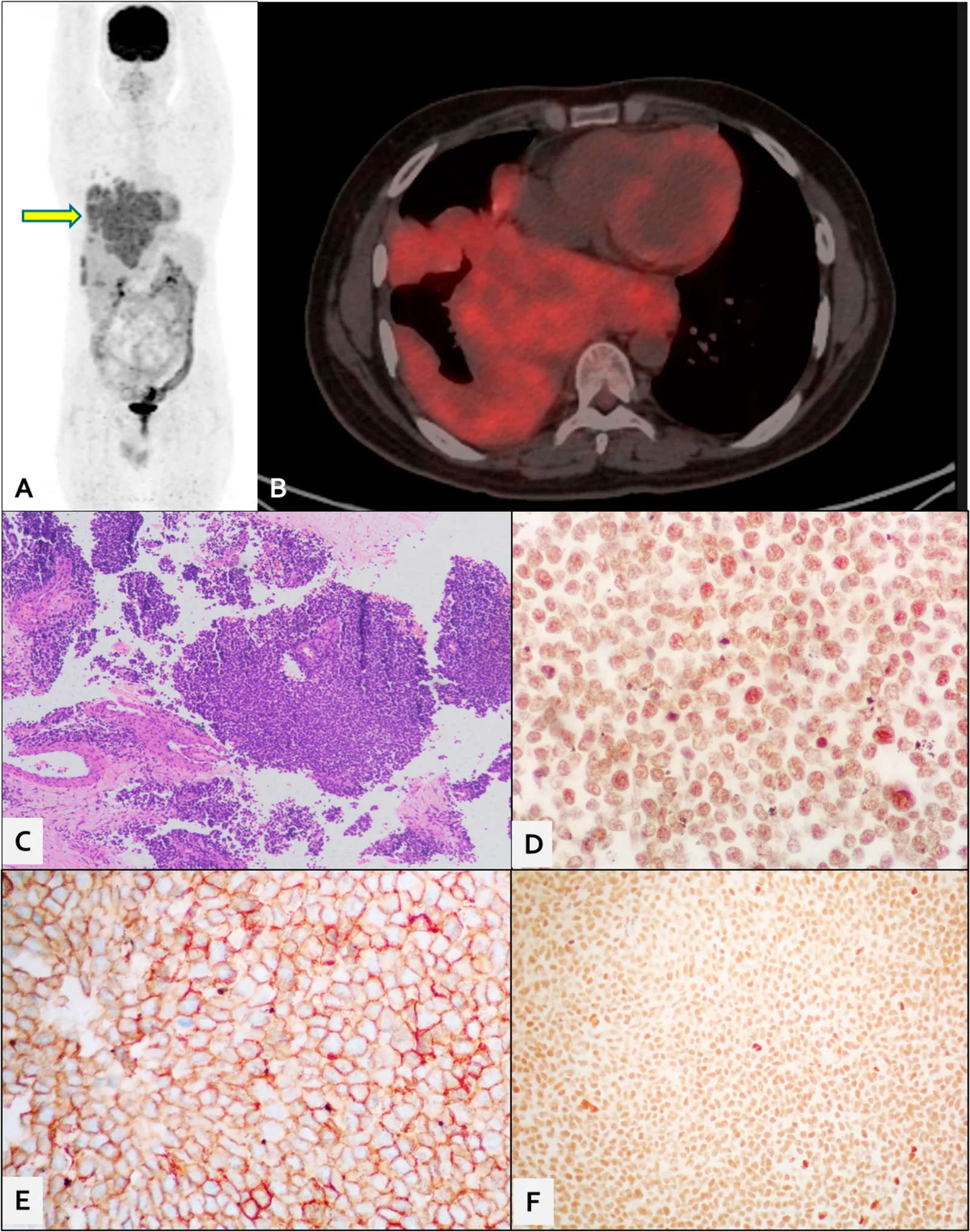
(**A**, **B**) Pre-treatment PET CT scan shows FDG avid left hemithoracic mass with areas of necrosis: 188 × 151 × 249 mm, SUVmax 7.53 (arrow). Left lung is not distinctly visualized. (**C**) Biopsy shows sheets of tumor cells with areas of necrosis, [H&E, 20X]. (**D**) On immunohistochemistry, tumor cells are positive for NKX2.2. (**E**) Complete membranous positivity is noted for CD99. (**F**) Fli-1 is positive in the tumor cell nuclei

Bone involvement was uniformly absent on computed tomography (CT) and magnetic resonance imaging (MRI). Among the 13 cases in our series, positron emission tomography (PET) imaging was available for 11 patients, all of which demonstrated exclusive pulmonary involvement without evidence of osseous or extrapulmonary disease. [Figures 1A and B and 2A and B].

The prototypical histopathological profile of the tumors in our series was characterized by cohesive sheets of small, round to oval neoplastic cells exhibiting centrally placed round to oval nuclei, scant basophilic cytoplasm, and finely dispersed, granular chromatin [Figs. 1C and 2C]. Distinct neuroectodermal differentiation, in the form of Homer Wright pseudorosettes or Flexner–Weinstein rosettes, was discernible in 23.1% of cases. Foci of coagulative necrosis and intratumoral hemorrhage were also identified in a subset of specimens.

Immunohistochemically, all assessable tumors demonstrated diffuse, strong membranous immunoreactivity for CD99 (10/10; 100%), with concurrent nuclear positivity for NKX2.2 (9/9; 100%) and FLI1 (6/6; 100%). Conversely, thyroid transcription factor-1 (TTF-1), cytokeratins, and desmin exhibited absent to negligible staining [Figs. 1D and 2D–F]. Co-expression of NKX2.2 and CD99 was observed in six cases, whereas two cases demonstrated triple-marker positivity for CD99, NKX2.2, and FLI1 [Table 2].

**Table 2:** Immunohistochemistry profile of primary pulmonary ewing’s sarcoma cases (*n* = 13)

Case no	NKX2.2	CD99	Fli-1	CD56	AE1/AE3	LCA	Desmin	Synapto	Chromo	TTF1
1	+	+	NA	NA	-	NA	-	+ (W)	NA	NA
2	+	+	+	NA	-	-	-	NA	NA	NA
3	+	+	NA	NA	-	-	-	NA	NA	NA
4	+	+	NA	NA	-	NA	NA	NA	NA	NA
5	NA	+	+	+	-	NA	NA	+ (F)	-	-
6	NA	+	+	NA	-	-	-	NA	NA	NA
7	+	+	NA	+	+ (dot like)	NA	NA	+	NA	NA
8	+	+	+	-	-	-	-	NA	NA	NA
9	NA	+	+	+	-	-	-	NA	NA	NA
10	NA	+	+		+ (dot like)	-	-	-	NA	NA
11	+	NA	NA	NA	-	-	-	NA	NA	NA
12	+	NA	NA	+ (patchy)	-	-	-	NA	NA	NA
13	+	NA	NA		-	-	NA	NA	NA	NA

*Synapto- Synaptophysin, Chromo- Chromogranin, NA- not available

At initial presentation, the majority of tumors measured greater than 10 cm in maximum dimension. Metastatic disease was documented in 46.2% of patients (6/13), with skeletal metastases developing later in the disease course in 38.5% (5/13). Systemic chemotherapy (CT) was the primary therapeutic modality, administered to 69.2% of patients, while smaller subsets received multimodal regimens comprising CT with radiotherapy (RT) (2/13) or CT with surgical resection (1/13) [Table 3].

**Table 3:** Treatment details and follow up status of the cases [*n* = 13]

Case no.	Treatment	Status at last follow up
1	CT + RT	Dead
2	CT	Dead
3	CT	Dead
4	CT	Progressive disease
5	CT + RT	Progressive disease
6	Surgery, CT	Alive with stable disease
7	CT	Progressive disease
8	CT	Alive with stable disease
9	CT	Progressive disease as metastasis
10	CT	Alive with stable disease
11	CT	Alive with stable disease
12	CT	Alive with stable disease
13	CT	Alive with stable disease

* CT- Chemotherapy, RT- Radiotherapy

Fluorescence in situ hybridization (FISH) analysis was undertaken in cases lacking classic histomorphological hallmarks, thereby presenting a diagnostic challenge. EWSR1 gene rearrangement was identified in four of six cases. The absence of a detectable translocation in one case was likely attributable to suboptimal tissue fixation in the externally processed specimen, whereas the inconclusive result in another case was plausibly due to extensive tumor necrosis, leaving insufficient viable neoplastic cells for reliable evaluation.

**Fig. 3:**
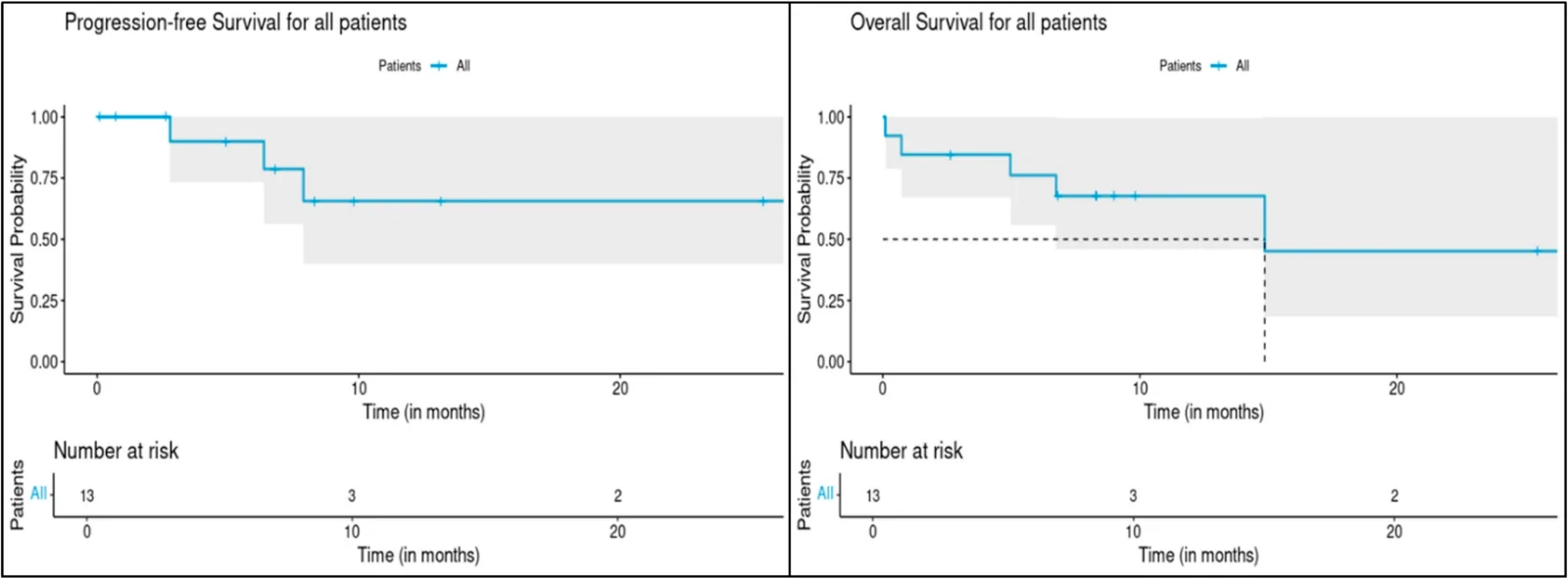
Follow up data of the patients

A total of 13 patients were included in the present analysis, of whom three experienced disease progression, while the remaining ten were either alive or lost to follow-up at the time of data cut-off. The mean progression-free survival (PFS) was 19 months (95% CI: 12.9–26.1 months) [Figure 3], with a 2-year disease-free survival rate of 65.6 ± 16.4% by using Kaplan–Meier method [Supplementary Table 4].

During the study period, five patients succumbed to the disease. The mean overall survival (OS) was 13 months (95% CI: 9.7–23 months), while the median OS was 15 months [Figure 3]. The estimated 2-year overall survival rate for the cohort was 45.1 ± 20.5% [Supplementary Table 5] by using Kaplan–Meier method.

## Discussion

Primary pulmonary Ewing sarcoma (PPES) represents an exceptionally uncommon subset within the Ewing sarcoma family, a group of aggressive small round cell tumors of presumed neuroectodermal origin. While osseous Ewing sarcomas are extensively characterized, extraosseous variants, particularly PPES remain poorly elucidated due to their rarity. Given the rarity of primary pulmonary primitive neuroectodermal tumors (PNETs), comprehensive clinical, radiological, and pathological assessment is imperative to exclude the possibility of metastatic disease from an extrapulmonary primary site. Clinical presentation is often nonspecific, with many patients presenting with a painful chest wall mass and systemic symptoms such as fever and malaise [7].

In this study of 13 histologically confirmed cases, we evaluated clinicopathological, immunohistochemical, and cytogenetic features, adding valuable data to the limited knowledge on this rare malignancy.

A marked male predominance (69.2%) and a broad age distribution (8–63 years) were noted in our cohort, consistent with Applebaum et al. [4], who reported that patients with extraskeletal Ewing sarcoma (EES) tend to present at an older age and lack the gender or racial predilection characteristic of the skeletal subtype. Most tumors were large (> 10 cm in 84.6%), in line with Purkayastha et al. [8], who documented that most lesions surpassed 9 cm in greatest diameter. Likewise, Tsetsou et al. [9] described a PPES case with a colossal 21 × 16 cm mass, underscoring the propensity for delayed diagnosis and substantial tumor burden at presentation in this rare entity.

The radiologic characteristics of tumors within the Ewing sarcoma (EWS) family are well described; however, imaging data specific to primary pulmonary Ewing sarcoma (PPES) remain scarce. When present, Radiologically, PPES typically appears on CT as a well-defined solitary mass with heterogeneous attenuation; calcifications and pleural effusions are occasional findings, while direct invasion of adjacent structures is rare [10]. Bone marrow involvement—a hallmark of osseous Ewing sarcoma—is generally absent in PPES and was not detected by FDG-PET in our patients, corroborating prior observations of Shet et al. [10], who emphasized that marrow infiltration is generally absent in pulmonary cases and can be reliably ruled out using FDG-PET.

Histopathologically, all tumors of this study showed typical small round cell morphology with necrosis and hemorrhage; Homer Wright rosettes were present in 23.1%, consistent with the neuroectodermal features described by Hammar et al. [11], who first delineated the neuroectodermal spectrum of such pulmonary neoplasms.

Immunohistochemically, all tested tumors exhibited diffuse membranous CD99 positivity (100%), with NKX2.2 expression detected in all nine evaluated cases (100%) and FLI1 positivity confirmed in all six tested cases (100%) [Supplementary Table 4]. This immunoprofile is highly supportive of Ewing sarcoma and aligns with the observations of Weissferdt and Moran [12], who similarly documented universal CD99 expression in their series of six PPES cases. Thyroid transcription factor-1 (TTF-1), desmin, and cytokeratin were uniformly negative, whereas synaptophysin demonstrated focal reactivity in a minority of cases. This finding parallels the variability noted by Fedeli et al. [6], where neuroendocrine marker expression was reported in 64.9% of cases, underscoring the potential diagnostic pitfall in distinguishing PPES from morphologically similar small round blue cell tumors, particularly small cell lung carcinoma.

Cytogenetically, fluorescence in situ hybridization (FISH) for EWSR1 rearrangement was performed in six diagnostically challenging cases, of which four (66.7%) were positive for definitive fusion. The identification of the EWSR1–FLI1 fusion, regarded as a molecular hallmark of Ewing sarcoma, is of critical diagnostic significance, as emphasized by Sankar et al. [2]. Our lower-than-expected positivity rate likely reflects technical limitations, including suboptimal tissue preservation and insufficient viable tumor cells in two biopsy-derived specimens, a limitation similarly highlighted by Zhang et al. [13], in their evaluation of molecular testing accuracy.

In this study, the majority of patients (69.2%) received systemic chemotherapy, while a smaller subset underwent multimodal therapy incorporating surgery and/or radiotherapy [Supplementary Table 5]. This treatment distribution parallels the findings of Sobh et al. [14] and Chen et al. [15], who reported improved survival with combined surgical resection and chemotherapy. Notably, only one patient in our series was suitable for surgical intervention, reflecting the frequent inoperability of PPES due to advanced disease at diagnosis. Neoadjuvant chemotherapy is typically employed to achieve tumor downstaging and facilitate resection, while adjuvant chemotherapy and radiotherapy have demonstrated partial therapeutic benefit in select cases [16]– [17].

Our clinical outcome analysis demonstrated a mean progression-free survival (PFS) of 19 months and a mean overall survival (OS) of 13 months. These metrics are comparable to those reported by Fedeli et al. [6], who documented a mean survival of 11.5 months and a mortality rate of 38.8%. Weissferdt and Moran [12] noted occasional long-term survivors, particularly among patients with resectable, early-stage disease, suggesting a markedly improved prognosis in this subgroup. Despite substantial therapeutic advances in non-small cell lung cancer (NSCLC)—including the advent of targeted EGFR/ALK inhibitors and immune checkpoint inhibitors—such strategies have no validated role in PPES. Ewing sarcoma remains a biologically and clinically distinct entity, and no targeted or immunotherapeutic agents are currently approved for this tumor subtype [18].

## Conclusion

The diagnosis of primary pulmonary Ewing sarcoma (PPES) poses a substantial challenge, as imaging alone is rarely definitive given the tumor’s extreme rarity and nonspecific radiologic features. As a malignant small round cell neoplasm, PPES requires meticulous histopathological assessment, with diagnosis supported by a characteristic immunophenotypic profile including diffuse NKX2.2 expression and confirmed by demonstration of EWSR1 gene rearrangement through fluorescence in situ hybridization (FISH), reverse transcription polymerase chain reaction (RT-PCR), or next-generation sequencing (NGS). Establishing an accurate diagnosis is critical for guiding optimal management, for which the current standard of care involves neoadjuvant chemotherapy followed by surgical resection.

## Supplementary Information

Below is the link to the electronic supplementary material.

Supplementary Material 1

## References

[1] Takahashi D, Nagayama J, Nagatoshi Y, Inagaki J, Nishiyama K, Yokoyama R et al (2007) Primary Ewing’s sarcoma family tumors of the lung: a case report and review of the literature. Jpn J Clin Oncol 37:874–7. 10.1093/jjco/hym108 17998263 10.1093/jjco/hym108

[2] Sankar S, Lessnick SL (2011) Promiscuous partnerships in ewing’s sarcoma. Cancer Genet 204(7):351–365. 10.1016/j.cancergen.2011.07.008 21872822 10.1016/j.cancergen.2011.07.008 PMC3164520

[3] Van den Berg H, Heinen RC, van der Pal HJ, Merks JH (2009) Extra-osseous Ewing sarcoma. Pediatr Hematol Oncol 26:175–85. 10.1080/08880010902855581 19437320 10.1080/08880010902855581

[4] Applebaum MA, Worch J, Matthay KK, Goldsby R, Neuhaus J, West DC, Dubois SG (2011) Clinical features and outcomes in patients with extraskeletal Ewing sarcoma. Cancer 117:3027–32. 10.1002/cncr.25840 21692057 10.1002/cncr.25840 PMC3135782

[5] Pankratjevaite L, Eskandarani HA, Lizdenis P, Saladzinskas Z (2022) Challenges in diagnosing an extraosseous Ewing sarcoma: a case report. Int J Surg Case Rep 91:106708. 10.1016/j.ijscr.2021.106708 35030406 10.1016/j.ijscr.2021.106708 PMC8760346

[6] Fedeli MA, Marras V, Fara AM, Deiana A, Lobrano R, Cossu A, Paliogiannis P (2023) Primary Ewing sarcoma of the lung: a systematic review of the recent literature. Ann Diagn Pathol 65:152152. 10.1016/j.anndiagpath.2023.152152 37149954 10.1016/j.anndiagpath.2023.152152

[7] Dong M, Liu J, Song Z, Li X, Shi T, Wang D et al (2015) Primary multiple pulmonary primitive neuroectodermal tumor: case report and literature review. Medicine (Baltimore) 94:e1136. 10.1097/MD.0000000000001136 26166119 10.1097/MD.0000000000001136 PMC4504587

[8] Purkayastha A, Pathak A, Sharma N, Viswanath S, Dutta V (2016) Primitive neuroectodermal tumor of lungs in adults: a rare series of three cases treated with upfront chemo-radiation. Transl Lung Cancer Res 5:350–355. 10.21037/tlcr.2016.06.03 27413716 10.21037/tlcr.2016.06.03 PMC4931133

[9] Tsetsou I, Moschouris H, Spanomanolis N, Soumpourou E (2022) Ewing sarcoma of the lung: imaging of a rare tumor. Cureus 14(12):e32395. 10.7759/cureus.32395 36636530 10.7759/cureus.32395 PMC9830843

[10] Shet N, Stanescu L, Deutsch G (2013) Primary extraosseous Ewing sarcoma of the lung: case report and literature review. Radiol Case Rep 8(2):832. 10.2484/rcr.v8i2.832 27330628 10.2484/rcr.v8i2.832 PMC4900125

[11] Hammar S, Bockus D, Remington F, Cooper L (1989) The unusual spectrum of neuroendocrine lung neoplasms. Ultrastruct Pathol 13:515–560. 10.3109/01913128909074534 2552631 10.3109/01913128909074534

[12] Weissferdt A, Moran CA (2012) Primary pulmonary primitive neuroectodermal tumor (PNET): a clinicopathological and immunohistochemical study of six cases. Lung 190(6):677–683. 10.1007/s00408-012-9405-9 22802134 10.1007/s00408-012-9405-9

[13] Zhang J, Dong A, Cui Y, Wang Y (2019) FDG PET/CT in a case of primary pulmonary ewing sarcoma. Clin Nucl Med 44:666–668. 10.1097/RLU.0000000000002659 31274619 10.1097/RLU.0000000000002659

[14] Sobha E, El-Sheshtawy WH, Anis SE (2017) Primary pulmonary extraskeletal ewing sarcoma/primitiveneuroectodermal tumor: two case reports. Egypt J Bronchol 11:161–164. 10.4103/ejb.ejb_48_16

[15] Chen J, Yuan T, Liu X, Hua B, Dong C, Liu Y et al (2019) Ewing’s sarcoma/peripheral primitive neuroectodermal tumors in bronchus. Am J Med Sci 357:75–80. 10.1016/j.amjms.2018.08.009 30314832 10.1016/j.amjms.2018.08.009

[16] Sohn AJ, Lang B, McCarroll M, Agarwal A (2020) Primary pulmonary ewing sarcoma/peripheral primitive neuroectodermal tumor. Proc (Bayl Univ Med Cent) 33:646–648. 10.1080/08998280.2020.1798723 33149376 10.1080/08998280.2020.1798723 PMC7590833

[17] Tungate RM, Lara K, Patel D, Fedenko A, Hu J (2022) Remission of a primary, recurrent thoracic Ewing sarcoma in a 74-year-old woman. Rare Tumors 14:20363613221110836. 10.1177/20363613221110836 35813490 10.1177/20363613221110836 PMC9260570

[18] Putzu C, Canova S, Paliogiannis P, Lobrano R, Sala L, Cortinovis DL et al (2023) Duration of immunotherapy in non-small cell lung cancer survivors: a lifelong commitment? Cancers (Basel) 15(3):689. 10.3390/cancers15030689 36765647 10.3390/cancers15030689 PMC9913378

