# Supplementary material for: Primary Ewing Sarcoma of the Lung: Integrative Analysis of Clinical, Histopathological, Immunohistochemical, and Cytogenetic Features with Literature Review
Source: Indian J Surg Oncol. 2026 Jan 5;17(8):1877–85. doi: 10.1007/s13193-025-02480-9 (PMC13518683; doi:10.1007/s13193-025-02480-9)
Supplement: Supplementary file 1 [file 13193_2025_2480_MOESM1_ESM.docx]

Table 4: Comparison of immunohistochemistry profile with the published cases

| **Author** | **Year** | **NKX2.2** | **CD99** | **Vimentin** | **S100** | **Desmin** | **Synapto** | **NSE** | **Chromo** | **TTF-1** | **PanCK** | **EWSR1-**  **FLI-1** |
| --- | --- | --- | --- | --- | --- | --- | --- | --- | --- | --- | --- | --- |
| **Weissferdt et al[12]**  **(6 cases)** | 2012 | NA | 6 + | 6 + | + | NA | 6 − | 6 + | 6 − | NA | NA | 2 +, 4 NA |
| **Purkayastha et al [8]**  **(3 cases)** | 2016 | NA | + | + | NA | NA | + | + | + | − | − | NA |
|  |  |  | + | + | NA | − | + | + | + | − | − | NA |
|  |  |  | + | + | + | NA | + | + | + | − | − | NA |
| **Sobha et al [14]**  **(2 cases)** | 2016 | NA | + | NA | NA | NA | NA | NA | NA | − | − | NA |
|  |  | NA | + | NA | NA | NA | NA | NA | NA | − | − | NA |
| **Sohn et al [19]** | 2020 | NA | + | NA | NA | NA | NA | NA | − | − | − | + |
| **Tsetsou et al [9]** | 2022 | NA | + | + | NA | − | + | NA | NA | NA | − | + |
| **Tungate et al [17]** | 2022 | NA | + | NA | NA | − | − | NA | − | − | NA | + |
| **Zhang et al [18]** | 2022 | NA | + | NA | NA | NA | − | − | − | − | − | + |
| **Present study**  **(13 cases)** | 2024 | 9+ | 8+ | 1+ | NA | - | 2+  (focal) | - | - | - | 2+ (dot like) | NA |

Table 5: Comparison of disease extension at diagnosis and overall prognosis with the published cases

| **Author(s)** | **Tumor size at diagnosis** | **Treatment** | **Prognosis** |
| --- | --- | --- | --- |
| **Weissferdt et al [12]**  **(6 cases)** | 5 cm mass | S | NA |
|  | 4 cm mass | S + CT | DOD at 24 m. |
|  | 5 cm mass | S + CT | DOD at 36 m. |
|  | 6 cm mass | S + CT | DOD at 54 m. |
|  | 6 cm mass | S | NA |
|  | 9.6 cm mass | CT + S + CT | FFD at 11 m. |
| **Purkayastha et al [8]**  **(3 cases)** | 9.3 × 8.3 × 9.4 cm mass | CT + RT | SD at 6 m. |
|  | 11.3 × 11 × 10 cm mass | CT + RT | SD at 6 m. |
|  | 10.5 × 9.3 × 9.8 cm mass | CT + RT | SD at 6 m. |
| **Sobha et al [14]**  **(2 cases)** | 9 × 10 cm mass | CT + S | FFD at 16 m. |
|  | 17 × 17 × 9.5 cm mass | CT + S | FFD at 35 m. |
| **Chen et al [15]** | 3.7 × 3.7 × 6.4 cm mass | S + CT | FFD at 18 m. |
| **Tsetsou et al [9]** | 21 × 16 cm mass + liver metastasis | CT | DOD at 2 m. |
| **Tungate et al [17]** | 10 × 16.4 × 15.6 mass | CT + S + RT | SD at 28 m. |
| **Zhang et al [18]** | 7.7 × 6.3 hilar mass | CT + S | FFD at 5 m |
| **Present study**  **(13 cases)** | ≤10 cm – 2, >10 cm- 11 | CT- 10 cases  CT+RT- 2 cases  CT+S- 1 case | PFS 19 m  OS 13 m |

- S- Surgery, CT- Chemotherapy, RT- Radiotherapy, DOD- Died of disease, FFD- Free from disease, SD- Stable disease, PFS- Progression free survival. OS- Overall survival.
